# PI3K-PANK4: a new target for de novo synthesis of coenzyme A

**DOI:** 10.1186/s43556-022-00094-z

**Published:** 2022-11-04

**Authors:** Jizhong Guan, Long Zhang, Fangfang Zhou

**Affiliations:** 1 School of Medicine, Zhejiang University City College, Hangzhou, 310015 Zhejiang China; 2 Institutes of Biology and Medical Science, Soochow University, Suzhou, 215123 China; 3 MOE Laboratory of Biosystems Homeostasis and Protection and Innovation Center for Cell Signaling Network, Life Sciences Institute, Zhejiang University, Hangzhou, 310058 China

In a recent study published in *Nature*, Dibble et al. discovered that growth factor-induced phosphoinositide-3-kinase (PI3K) activation led to AKT phosphorylation and activation. PANK2 and PANK4 were identified as AKT substrates, and the unique PANK4 related 4′-phosphopantetheine directly limited coenzyme A (CoA) synthesis. With the loss of PANK4 phosphatase activity, CoA synthesis was restored [1] (Fig. 1).

**Fig. 1:**
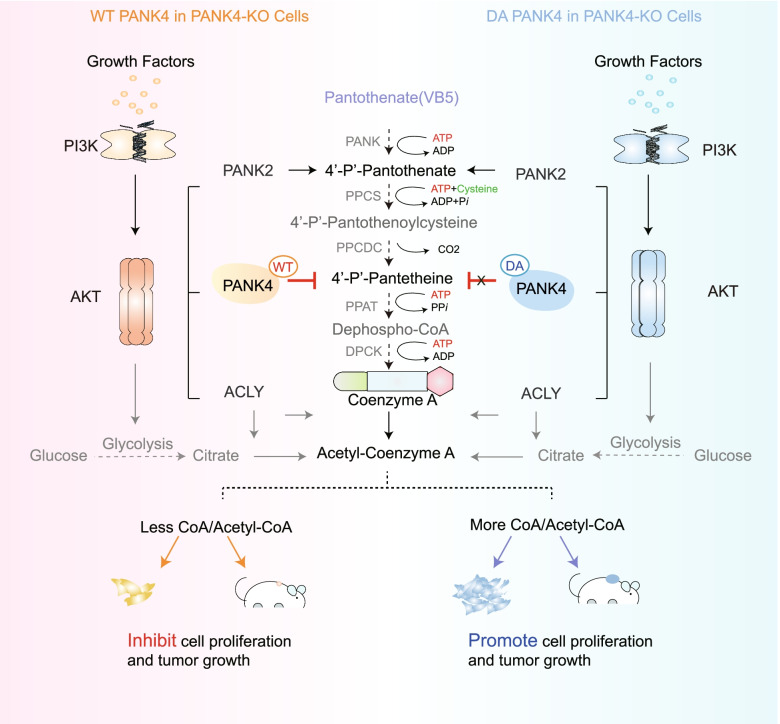
PI3K-PANK4 axis inhibits CoA and Acetyl-CoA synthesis. The basic ingredients vitamin B5, cysteine, and ATP are converted into 4'-P-Pantothenate, 4'-P-Pantothenoylcysteine, 4'-P-Pantetheine, and lastly CoA and acetyl-CoA by an enzymatic reaction. WT PANK4 was responsive to 4'-P-Panthionine and inhibited coenzyme A and acetyl-CoA production, thereby inhibiting cell proliferation and tumor growth,whereas DA PANK4 was not

As aubiquitous intracellular cofactor, CoA expression has been linked to extracellular stimulation, metabolite production, and human diseases. Previous studies have demonstrated that organisms require pantothenate (vitamin B5, VB5), cysteine, and ATP to synthesize CoA via five conserved enzymatic steps. First, pantothenate isphosphorylatedby PANK to 4′-phosphopantothenate (4′-P-Pantothenate),which issubsequently condensedwithcysteine by 4′-P-Pantothenoylcysteine synthase (PPCS) to form 4′-P-Pantothenoylcysteine, decarboxylated by 4′-P-Pantothenoylcysteine decarboxylase (PPCDC) toform 4′-P-Pantetheine, converted to dephospho-CoA by phosphopantetheine adenylyltransferase (PPAT), and, finally, phosphorylated at the 3-OH of the ribose by dephospho-CoA kinase (DPCK) to form CoA [2]. CoA is essential for cellular metabolism and is closely related to liver disease [3].

Extracellular growth factors, hormones, and associated stimulatory factors activate PI3K, which in turn activates AKT via phosphatidylinositol (3,4,5)-trisphosphate, phosphoinositide-dependent protein kinase 1, and the rapamycin complex (mTORC). The most crucial component of cell signaling, PI3K–AKT, is required for cell growth, proliferation, and differentiation [4]. AKT has long been implicated in the synthesis of acetyl-CoA, including by glycolysis and ATP-citrate lyase (ACLY), which are essential for the synthesis of acetyl-CoA fromcitrate and CoA [5]. However, the role of PI3K in metabolism has not yet been elucidated; the authors investigated the mechanism of action of PI3K in the synthesis of CoA, including acetyl-CoA, and their findings have opened up potential opportunities for the treatment of metabolic illnesses in the future.

The authors first discovered that activation of PI3K and AKT can increase CoA synthesis. Based on isotope (^13^C_3_^15^N_1_)-labeled VB5, the researchers discovered that insulin-stimulated labeling of CoA was completely inhibited by a PI3K inhibitor, while VB5 levels were increased. Growth factor stimulation also led to an increase in the quantity of CoA and acetyl-CoA, which PI3K inhibition also blocked, but did not prevent mitogen-activated protein kinase from being activated by growth factor stimulation. The constitutively active point mutant of PI3Kα, H1047R, can also directly increase CoA levels in the absence of external stimuli, and the authors ruled out additional processes that could contribute to the elevation of CoA, such as acyl-CoA deacylation, depletion, or degradation. This implies that PI3K encourages the de novo production of CoA and acetyl-CoA by VB5.

AKT, a key PI3K effector, produced similar results.Furthermore,AKT was inhibited by downstream mTORC1, thereby increasing CoA synthesis. The authors found that AKT inhibitors continued to work even when ACLY was inhibited, suggesting that AKT can independently regulate CoA synthesis.

Next, the authors discovered that PANK2 and PANK4 are AKT substrates by identifying the consensus AKT substrate sequence and antibody. The function of PANK4 is unknown, whereas PANK2 has long been recognized as a significant kinase in the initial stage of CoA production, pantothenate-kinase-associated neurodegeneration (PKAN) may be caused by reduced levels of CoA due to mutations in PANK2. The researchers discovered that tumors from *Pik3ca*^*p.H1047R*^ tumor-bearing mice treated with a PI3K inhibitor exhibited PANK4 phosphorylation and tumor growth in a PI3K-dependent manner; similar effects were observed in the skeletal muscle of non-tumor-bearing mice and in IGF-1–stimulated cells. Purified AKT can phosphorylate Ser169 and Ser189 of purified PANK2 and Thr406 of purifiedPANK4 in vitro. As a result, the authors suggested that PANK2 and PANK4 may play significant roles in facilitating the effect of AKT on CoA abundance.

The authors knocked out PANK2 and PANK4 expression in *AKT*^*p.E17K/*+^ knock-in cells to ascertain their function in AKT-mediated CoA synthesis. As expected, PANK2 knockout suppressed CoA expression. Interestingly, PANK4 had the opposite effect, as its absence unexpectedly increased CoA synthesis. The scientists then re-expressed wild-type (WT) PANK4, phosphorylation-deficient PANK4 (T406A), or phospho-PANK4 (T406E) in PANK4-knockout cells to corroborate this finding. Compared with WT PANK4, T406A reduced CoA production and inhibited cell growth. T406E was substantially less effective than T406A in reducing the buildup of total CoA observed following ACLY inhibition.Collectively, the inhibitory effect of PANK4 was lessened by AKT phosphorylation of Thr406, which increased CoA production and cell proliferation.

Finally, the authors looked to determine the mechanism by which PANK4 influences CoA levels. Their first finding was that PANK4 and its homologues shared the same phosphatase activity.Next, they identified that Asp623 and Asp659 were required for PANK4 phosphatase activity. Recent findings indicate that the high activity of PANK4 is biased toward 4'-phosphopantetheine rather than toward 4'-phosphopantothenate, which may represent a novel finding regarding the function of PANK4.The authors observed that kinase-inactive PANK4 (D623A and D659A) lost its inhibitory effect on CoA synthesis when re-expressed in PANK4-knockout cells, and metabolomic data further suggested that CoA abundance is dependent on PANK4 phosphatase activity. Researchers have also discovered that PANK4, which depends on its phosphatase activity, causes anomalies in the synthesis of fatty acids and the assembly of lipids as well as insufficient mitochondrial respiration and decreased histone acetylation, all of which inhibit cell growth and cancer.

PI3K inhibitors have been attractive anticancer targets, but PI3K inhibitors alone have limited efficacy and relatively high incidence of side effects. The findings of the Dibble et al. allow us to consider increasing the phosphatase activity of PANK4 to achieve tumor suppression, so the combination of PI3K inhibitors and PANK4 activators is also worth discussing. Overall, PANK4 regulates tumor growth through the synthesis of CoA, providing a new idea for PI3K to treat tumors.

Regarding the study of cell metabolism and related diseases, an increasing number of scientists have focused on CoA despite the facts that the PI3K–PANK4 axis plays a clear role in CoA production and several inhibitors have been demonstrated to efficiently inhibit the PI3K-Akt signaling pathway. Additionally, a range of pathways mediated by PI3K have varied effects on CoA production, which can proceed via other pathways. CoA degradation is still subject to regulation, and the specific mechanisms remain unknown; this aspect deserves further research and discussion. Noting that compensation from other signaling pathways in vivo still needs to be avoided, a multi-drug combination approach may be the best choice for treating metabolic diseases; however, druggability and clinical validation will be required.

In conclusion, the authors proposed a novel strategy to modulate CoA, which they believe may be beneficial for patients with PKAN or PI3K-dependent malignancies. It is worth mentioning that the possibility of PANK4 as a single drug treatment is also worth exploring, as its effect on the treatment of CoA-induced liver diseases is promising. The discovery of PANK4 provides strong promise for the treatment of CoA-related diseases; however, we have only uncovered the tip of the iceberg at this point.

## Data Availability

Not applicable.
